# Quantitative Estimation of Low-Abundance Targets in Engineered Systems and Environmental Samples: Comparative Study Between Droplet Digital PCR and Real-Time PCR

**DOI:** 10.3390/microorganisms13112426

**Published:** 2025-10-23

**Authors:** Alessia Ayala Alban, Barbara Tonanzi, Simona Crognale, Francesca Di Pippo, Simona Rossetti

**Affiliations:** 1 Water Research Institute, National Research Council of Italy, CNR-IRSA, Via Salaria Km 29.300, Monterotondo, 00015 Rome, Italy; 2 National Biodiversity Future Center (NBFC), Piazza Marina 61, 90133 Palermo, Italy

**Keywords:** droplet digital PCR, absolute quantification, Real-Time PCR, ammonia-oxidizing bacteria (AOB), freshwater and marine samples, activated sludge, granular biomass

## Abstract

Real-Time PCR (qPCR) is an extensively used biomolecular tool for the detection and quantification of nucleic acids for a variety of applications, spanning from clinical to environmental settings. However, qPCR relies on an external calibration curve and can be susceptible to inhibition caused by pollutants that are commonly found in environmental samples. More recently, droplet digital PCR (ddPCR) was proven to be the method of choice for detection and quantification when a target is present at a low abundance. While it has been extensively utilized in clinical studies, only a small amount of data is available for complex samples, which are often characterized by a low target concentration and high abundances of non-target and PCR inhibitors. In this study, ddPCR and qPCR assays were performed on the same DNA serial dilutions with both Eva/SYBR Green and TaqMan chemistry. The comparative analysis was conducted on seven different samples taken from environmental and engineered settings. Ammonia-oxidizing bacteria (AOB) were chosen as the target, as they are ubiquitous and widespread and responsible for a fundamental environmental process in the global biogeochemical nitrogen cycle and in engineered settings such as wastewater treatment plants (WWPTs). ddPCR produced precise, reproducible, and statistically significant results in all samples, also showing an increased sensitivity to detecting AOB in complex samples characterized by low levels of the target and low target/non-target ratios.

## 1. Introduction

The real-time quantification of nucleic acids is fundamental for a wide range of applications, spanning from medical diagnostic purposes to biomonitoring of specific biological taxa or functional genes in industrial processes and environmental settings. In this context, the use of biomolecular tools (MBTs) has become of paramount importance due to their specificity and sensitivity [1]. Among the MBTs, Real-Time PCR (qPCR) has significantly improved and simplified the rapid quantification of nucleic acids and still represents a valuable tool for many scientists and users working in different disciplines and sectors. However, it is affected by a few critical issues that strongly limit its reliability and applicability under certain conditions, such as low target concentrations or low target/total genomic DNA ratios. Moreover, the measurement is indirect and relies on standard references to interpolate the quantification of the target. This may further introduce variability and error factors [2]. Moreover, qPCR is susceptible to the presence of inhibitors, which are often present in environmental samples [3]. This can be partly overcome by diluting the sample or purifying it. However, in the case of environmental samples, where DNA or target concentrations are often low, the dilution further reduces the target gene copy number per reaction, pushing it below the limit of detection of qPCR.

In recent years, digital droplet PCR (ddPCR) has emerged as a new third-generation quantification technology enabling the absolute quantification of nucleic acids independently of a standard reference material. In ddPCR, the reaction occurs in thousands of nanoliter-sized droplets obtained by water-in-oil emulsion. Each droplet acts as an individual microreactor which may contain zero, one, or a few copies of the target DNA sequence. During amplification, only droplets containing the target DNA will generate a fluorescence signal and will be counted as a positive reaction, as opposed to those that will not fluoresce (negative). After amplification, the droplets will be detected one by one, allowing for an absolute measure of the target DNA concentration by the statistic Poisson method [4].

Therefore, some of the main limitations of qPCR are overcome by ddPCR being capable of absolute quantification and making external calibrators unnecessary. Most importantly, the impact of contaminants on the amplification efficiency and the consequent need for dilution in low-concentration samples is reduced by partitioning the reaction into droplets and transforming the signal into binary scores [3,4]. Finally, partitioning reduces the reaction volume, thereby increasing the target concentration in each droplet and improving the limit of detection [5].

The high sensitivity, accuracy, and reproducibility of ddPCR have led to its widespread use in many fields of application, mainly in the medical diagnostic field [6,7] and food testing [8,9,10]. Very few examples of ddPCR application are reported for the analysis of environmental samples, and these are mostly focused on human health-related targets such as antibiotic resistance genes (ARGs) and pathogenic microorganisms/indicators [3,11,12,13,14].

The purpose of this study was to compare the efficacy and sensitivity of ddPCR with qPCR, using both SYBR/Eva Green and TaqMan chemistry, for the analysis of environmental and engineered settings. We have specifically evaluated if ddPCR assay is a viable tool to quantify low levels of a target in complex samples, which are typically characterized by the occurrence of high non-target abundance and PCR inhibitors, which often occur in environmental samples and in industrial biological processes, where the use of a robust, easy-to-apply, and rapid biomonitoring tool represents a crucial need. For the comparative study, ammonia-oxidizing bacteria (AOB) were chosen as the target, as they are ubiquitous and widespread in both marine and freshwater aquatic environments, as well widely found to be enriched in WWPTs with nitrogen removal.

## 2. Materials and Methods

### 2.1. Sampling and Storage

A total of seven samples were collected from distinct sources: 50 mL of activated sludge (AS) from a full-scale municipal wastewater treatment plant located in Rome (Italy), 50 mL of granular sludge taken from four laboratory-scale granular sludge reactors (named as R1, R2, R3, R4) operated for nitrogen removal and characterized by different total biomass concentrations and AOB/total bacteria ratios, 1 L of freshwater sample (FW) collected from a volcanic-origin lake situated among the Sabatini Mountains, Bracciano lake (Central Italy), and 1 L of seawater sample (SW) from the first inlet of Mar Piccolo, Taranto (Southern Italy). At least duplicate 2 mL aliquots of AS were centrifuged as previously described [15], and the resulting pellets were stored at −20 °C for DNA extraction. About 150 mg of wet granules were taken from each granular sludge reactor (R1–R4) and stored at −20 °C for further processing. For environmental samples, 250 mL and 650 mL of FW and SW, respectively, were immediately filtered by gentle vacuum (<0.2 bar) on polycarbonate filters (0.22 μm pore size, 47 mm diameter, Nuclepore) and stored in sterile Petri dishes at −20 °C until DNA extraction.

### 2.2. DNA Extraction

DNA extraction was performed using the DNeasy PowerSoil Pro Kit (QIAGEN, Milan, Italy), following the manufacturer’s instructions without modification. DNA concentration was measured using a NanoDrop 2000 spectrophotometer (Thermo Scientific, Monza, Italy). Purified DNA from each sample was eluted in 50 μL sterile Milli-Q and stored at −20 °C for further biomolecular analysis. DNA extracted from the samples analyzed in this study showed a 260/280 ratio in the range of 1.9–2.0, indicating a high-quality DNA template, whereas the 260/230 ratios were very low (in most cases < 0.7), suggesting the occurrence of inhibitors.

### 2.3. Primer and Probe

The primer set utilized targets ammonia-oxidizing bacteria (AOB); the forward primers CTO189fAB and CTO189fC were used in a 2:1 ratio with RT1r reverse primer, following the methodology previously described [16]. TMP1 probe was also utilized in the probe-based assay according to Hermansson et al. [16]. Total bacteria were estimated in each sample by SYBR Green qPCR targeting the 16S rRNA gene, following the procedures previously described [17]. The nucleotide sequences of primers and probes are provided in Table 1.

### 2.4. Annealing Temperature Optimization in ddPCR Assay

The optimal annealing temperature for the target gene amplification was determined through an initial in silico approach using a Tm calculator from Thermo Scientific, followed by experimental validation. PCR amplification was performed using DreamTaq Hot Start PCR Master Mix (2×) (Thermo Scientific) in a total reaction volume of 25 µL, consisting of 0.5 µM of forward and reverse primers, 2 µL of template DNA, and sterile MilliQ water to adjust the final volume. Thermal cycling was conducted in a Bio-Rad T100 thermocycler under the following conditions: 95 °C for 5 min, 35 cycles at 95 °C for 30 s, annealing in a range of different temperatures from 55.5 °C to 60.5 °C with a step difference of 0.5 °C for 30 s, 72 °C for 1 min, and a final cycle at 72 °C for 5 min. The resulting PCR products were visualized on a 2% agarose gel.

### 2.5. ddPCR Assay

ddPCR assays were carried out using both EvaGreen and TaqMan chemistry. EvaGreen reaction mixtures were prepared in 22 µL final volume: 11 µL of (2×) QX200 ddpCR EvaGreen Supermix (Bio-Rad, Hercules, CA, USA), 0.25 µM of forward and reverse primers, 2 µL of DNA, and nuclease-free water up to the final volume. In the TaqMan-based assay, each reaction included 0.9 µM of primers and 0.25 µM of probe, 11 µL of (2×) ddPCR Supermix for Probes (No dUTP), 2 µL of DNA, and nuclease-free water to reach the final volume of 22 µL. In both assay formats, a no-template control (NTC) was included in duplicate in every run by replacing the 2 µL of DNA template with nuclease-free water, serving to monitor reagent and cross-contamination. NTCs did not yield any positive droplets. When it happened on rare occasions, the run was repeated.

In both assays, the reaction droplets were prepared using an 8-channel droplet generation cartridge (Bio-Rad, USA) in combination with the QX200 Droplet Generator (Bio-Rad, USA). For each well, 20 μL reaction mix was combined with 70 μL of specific dPCR droplet generation oil (Bio-Rad, USA). The resulting water-on-oil emulsions were transferred to 96-well PCR plates and amplified in a T100 thermal cycler (Bio-Rad, USA).

For EvaGreen assays, the PCR was performed as follows: 5 min at 95 °C, 30 s at 95 °C for 40 cycles, and 1 min at 56 °C (ramping rate set to 2 °C/s).

For TaqMan assays, the PCR protocol was 10 min at 95 °C, and 40 cycles of the following: 30 s at 94 °C, 1 min at 56 °C, and 10 min at 98 °C. Following amplification, droplet fluorescence was read using the QX200 Droplet Reader (Bio-Rad, USA). Data analysis was performed using QuantaSoft™ software version 1.2 (Bio-Rad, USA), and the threshold was set manually. Samples with accepted droplets under 10,000 were not included in the analysis. All reactions were performed in duplicate.

### 2.6. qPCR Assay

SYBR Green and TaqMan qPCR assays were performed in CFX96 Real-Time PCR (Bio-Rad, USA). For SYBR Green qPCR, reactions were set up using 10 µL of (2×) SsoAdvanced Universal SYBR Green Supermix, 0.37 µM of each primer, 2 µL of DNA, and nuclease-free water up to final volume of 20 µL. The thermal cycling conditions were 95 °C for 3 min, followed by 35 cycles of 95 °C for 15 s and 60 °C for 30 s.

For TaqMan qPCR, the 20 µL reaction mix included 10 µL of (2×) SsoAdvanced Universal Probes Supermix, 0.9 µM of each primer, 0.25 µM of probe, 2 µL of DNA, and nuclease-free water to reach the final volume. Each qPCR run included NTC in triplicate. NTC reaction was prepared identically to the samples, but the 2 µL of DNA template was replaced with an equivalent volume of nuclease-free water. In both qPCR assays, NTCs showed no amplification signal (undetermined Ct values) up to 35 cycles, confirming the absence of detectable target DNA contamination in the reagents or workflow.

The thermal cycling procedure was 95 °C for 3 min, followed by 35 cycles of 95 °C for 15 s and 60 °C for 30 s. All reactions were performed in triplicate.

Standard curves for the absolute quantification were constructed by using the long amplicon method previously reported [18].

### 2.7. Dilution Series Preparation

The DNA samples were serially diluted immediately before the ddPCR and qPCR experiments. The dilution was chosen based on the initial concentration, measured with the NanoDrop 2000 spectrophotometer. At least duplicate dilution series of DNA were prepared separately for both techniques, and the same sample was used as the template for both ddPCR and qPCR assays in all experiments. Moreover, a no-template control was always analyzed, and all assays were performed by the same person.

For each ddPCR and qPCR dilution series, the log gene copy numbers per reaction and Ct (cycle threshold) values were, respectively, plotted against log of the DNA concentration.

### 2.8. Statistical Analysis

For each dilution series analyzed by both techniques with two different chemistries, copy numbers per reaction were transformed as log of copy numbers per microliter of reaction (ddPCR) or gene copies per microliter of extracted DNA (qPCR), and coefficient of determination (R2) was used to determine PCR efficiency. The coefficient of variation (CV), expressed as a percentage, was calculated as the ratio of the standard deviation to the mean. As a high CV value corresponds to a high dispersion around the mean, it was used to determine the repeatability and limit of quantification of each method. The lower and upper limits of the linear range were used to define the quantification range of each assay.

The principal component analysis (PCA), based on the correlation matrix, was performed using the software PAST (PAlaeontological STatistics, ver. 2.17) [19] and SigmaPlot (v. 14.0, Systat Software, San Jose, CA, USA). Prior to analysis, all variables, including gene copy numbers from qPCR (TaqMan, SYBR Green) and ddPCR (TaqMan, EvaGreen) and the AOB-to-total bacteria ratio, were log-transformed (log (X + 1)) to normalize the data distribution. PCA was used to visualize sample clustering and identify variables driving the separation. Major variance components explaining sample grouping were extracted. Differences among groups and techniques were statistically validated via a Permutational Multivariate Analysis of Variance PERMANOVA on the same dataset.

## 3. Results and Discussion

The comparative analysis was performed on seven different samples, characterized by various levels of complexity in terms of target concentration and low target/non-target ratio (% AOB/total bacteria; Table 2). As shown in Table 2, we examined both environmental samples (lake and seawater) and aggregated biomass composed of mixed microbial communities, selected from laboratory and full-scale wastewater treatment systems (R1–R4 and AS), with the objective to evaluate the efficacy and sensitivity of ddPCR in quantifying AOB. The outputs of ddPCR analysis were compared with those obtained on the same DNA dilutions by qPCR.

### 3.1. ddPCR Assay Optimization

The optimal annealing temperature that was previously reported and utilized for AOB quantification by qPCR [16] was re-evaluated to ensure correct specificity in the ddPCR assay. The optimum annealing temperature was determined by gradient PCR over an annealing temperature range of 55.5 °C and 60.5 °C (Figure S1 in Supplementary Information, SI). The optimal annealing temperature of 56 °C was chosen for further experiments and enabled reliable amplification and precise droplet partitioning, resulting in the generation of an average of over 16,000 droplets per assay.

Subsequently, the ddPCR assays were performed with various ad hoc dilutions for each sample. The sample dilution was previously shown to enhance the resolution of positive droplets in environmental samples [20] due to the reduction in potential inhibitors present in the samples. As it is not possible to know the most appropriate dilution in advance, a tailor-made approach for each individual sample was necessary, as detailed in Section 3.2.

### 3.2. Quantification Range and Assay Sensitivity

At least three dilution series of DNA extracted from the seven analyzed samples were prepared separately, as described in Section 2.7. To define and compare the quantification range of ddPCR and qPCR, the dilution series were run independently, with triplicate and duplicate reactions for qPCR and ddPCR, respectively.

**Figure 1:**
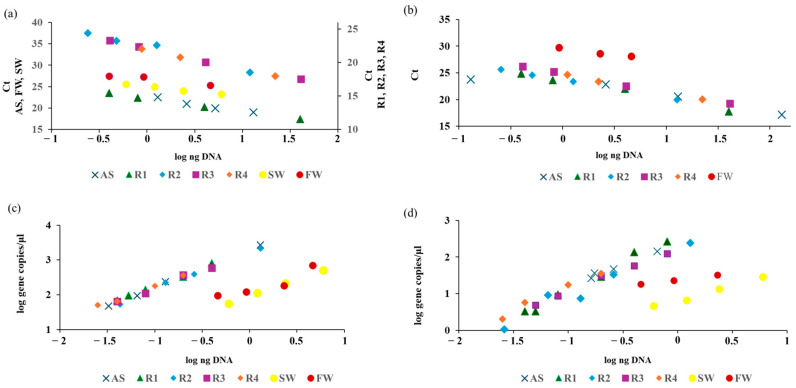
AOB quantification by (**a**) SYBR Green qPCR, (**b**) TaqMan qPCR, (**c**) EvaGreen ddPCR, and (**d**) TaqMan ddPCR on diluted DNA extracted from seven different samples. The R2 values from the linear regression analysis of each dilution series are reported in Table 3. AOB concentration, estimated by ddPCR (**c**,**d**), is expressed as log gene copies μL^−1^ reaction volume.

qPCR applied on AS and R1-R4 samples measured the AOB DNA over a wider dilution range (0.13–130 ng; Figure 1a,b) compared to ddPCR, which exhibited linearity in a narrow range (0.025–4.6 ng DNA; Figure 1c,d). However, qPCR failed at low target concentrations, especially when TaqMan chemistry was employed (Figure 1b). This was particularly evident for FW and SW samples, where already at ten-fold dilutions, no outputs were obtained.

At lower DNA dilutions, the ddPCR partitions became saturated and the gene copy numbers were out of the linearity range, whereas the target quantification at lower DNA concentrations were linearly correlated in all samples analyzed, including the ones characterized by a lower target/no target ratio. As shown in Table 3 and Table 4, the linear regression analysis of qPCR and ddPCR dilution series showed high linearity and slopes with R^2^ > 0.9, apart from FW (0.84) quantified by EvaGreen ddPCR. The coefficient of variation (CV) was below 17% in all samples analyzed by both techniques and chemistries (Table 3 and Table 4).

Overall, EvaGreen ddPCR reduced the mean CV by 13–82% compared to SYBR Green qPCR. On the other hand, the comparison of AOB quantification by TaqMan ddPCR and qPCR showed high variability in terms of performance: a greater precision of ddPCR was observed only for AS and FW samples (CV decreased, ranging from 24 to 74%), whereas qPCR was able to quantify the target on the two-fold diluted SW sample only. This finding could also be due to the high dilution used in ddPCR, which might have artificially reduced variation compared to less-diluted qPCR reactions.

The detailed quantification range estimated for each ddPCR and qPCR assay is reported in Table S1 in the Supplementary Information (SI). Overall, considering data from all samples within the linear dilution range, qPCR was able to detect AOB in a range of 7.2–8.85 × 10^5^ gene copies μL^−1^ reaction volume (corresponding to 0.92–130 ng DNA per reaction, respectively). Compared to qPCR, ddPCR showed higher sensitivity, being able to detect 1 copy μL^−1^ up to 8.58 × 10^2^ gene copies μL^−1^ reaction volume (corresponding to 0.026–1.3 ng DNA per reaction). This finding is in line with previous comparative studies performed on samples at known target concentrations [2] and on positive controls, followed by validation on environmental samples by ddPCR only [14].

### 3.3. Comparison of AOB Quantification by ddPCR and qPCR

**Figure 2:**
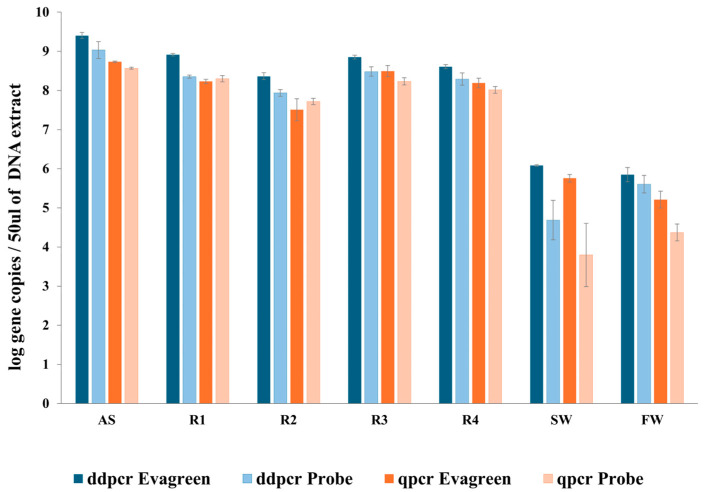
AOB quantification by ddPCR and qPCR with DNA staining (EvaGreen and SYBR Green) and probe-based chemistry on samples from seven different sources characterized by different target (AOB) concentrations and target/non-target ratios. Data are expressed as log of AOB gene copies/50 μL of DNA extracted. Only average data within the linearity range are reported, with the sole exception of TaqMan qPCR in the SW sample, where the analysis was performed only on the two-fold diluted sample due to a low target concentration. Error bars indicate standard deviation.

The mean AOB quantification performed by ddPCR and qPCR in EvaGreen/SYBR Green and Taqman chemistries is reported in Figure 2. Mean values are calculated using only the values estimated within the linearity range shown in Figure 1. Remarkably, numbers of the same order of magnitude were obtained for all samples analyzed, except for those characterized by lower target concentrations (SW and FW). In the latter case, ddPCR produced reliable results, estimated within a wider linearity dilution range compared to qPCR. Overall, the AOB estimates by both techniques using DNA intercalating fluorescent dyes were up to four-fold higher than those obtained with probe-based assays (Tables S2 and S3 in Supplementary Information, SI). Compared with Taqman outputs, a marked increase was specifically observed for SW analyzed by EvaGreen ddPCR (15-fold increase) and for FW and SW samples by SYBR Green qPCR (up to 48-fold increase). The observed discrepancy is likely due to the non-specific amplification that may occur when chemistries using a DNA intercalating dye (SYBR Green and EvaGreen) are employed for the analysis of complex environmental samples.

Similar increases were observed when the comparison was made between ddPCR and qPCR data obtained using the same chemistry. Compared with qPCR with the same chemistry, an up to 6-fold increase was observed for ddPCR data in all samples except for FW (17-fold increase in Taqman). Quantitative data from the different datasets, shown in Tables S1 and S2, were significantly different (*p* < 0.01).

The outputs clearly showed a qPCR AOB quantification of at least one order of magnitude lower with respect to ddPCR in the analysis of samples containing low target concentrations. This was particularly evident in assays performed with TaqMan qPCR (Tables S2 and S3).

Even though ddPCR has previously largely been considered as an elective quantitative tool in many medical and diagnostic applications, this is the first study analyzing its significant potential for the analysis of a set of different complex environmental samples, including those characterized by high concentrations of non-targets, as in the case of activated sludge and granular biomass (VSS up to 4.6 gL^−1^).

**Figure 3:**
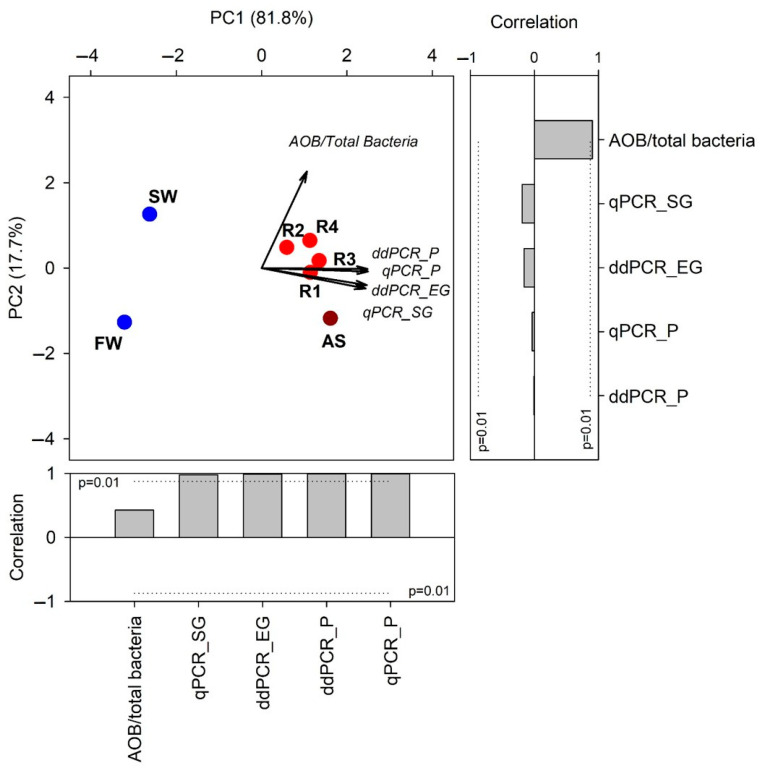
Principal component analysis (PCA) biplot representing the distribution of samples and variables. The vector length is proportional to the correlation between the corresponding parameter and the PCA axes 1 and 2. Barplots show the contribution of each variable (vector projection values), expressed as the correlation with the *x*- and *y*-axis.

The PCA, performed on normalized quantitative data, showed a well-defined structure in the dataset, with the first two principal components (PC1 and PC2) accounting for 99.5% of the total variance (PC1 = 81.8%; PC2 = 17.7%) (Figure 3). Samples clustered distinctly along PC1, clearly separating engineered systems (AS, R1–R4) from natural aquatic environments (FW, SW). This grouping was statistically supported by PERMANOVA (*p* < 0.05), indicating a significant difference in quantitative profiles between the two sample types.

Variables that were significantly correlated with PC1 and contributed most strongly to its variance, thus driving sample clustering, included gene copy numbers measured by both qPCR and ddPCR assays, as well as the AOB/total bacteria ratio. These findings suggest that the overall abundance of target genes is a key factor differentiating these sample types, likely reflecting the relative enrichment of AOB in engineered systems. Although PC2 explained a smaller portion of the variance, its association with the AOB/total bacteria ratio points to a secondary role of relative abundance in sample discrimination. The observed patterns likely reflect microbial community differences, such as community structures and levels of AOB enrichment between engineered and natural aquatic samples.

Interestingly, the variables related to gene copy numbers were closely associated based on their detection chemistry: those using a fluorescent probe (TaqMan) vs. those using an intercalating dye (SYBR Green and EvaGreen). This finding aligns with the study’s objective, demonstrating that the quantification methods (qPCR vs. ddPCR) and their underlying chemistries (TaqMan vs. intercalating dyes) significantly influence the resulting data and their relationships, supporting the need for a comparative analysis of these biomolecular tools in complex environmental matrices.

## 4. Conclusions

This study showed high sensitivity and precision of ddPCR in the analysis of complex samples from environmental and engineered settings, which have rarely been analyzed by this technique in previous investigations. ddPCR was shown to be a valid alternative to qPCR, being able to produce reliable and precise quantification of the target in a wide range of target concentrations, including low-abundance ones for which qPCR usually fails, or produces outputs characterized by high variability. Due to this unique capability, it allowed the precise and statistically significant quantification of AOB in all screened samples by circumventing, through high DNA dilution, the occurrence of potential inhibitors that often occur in environmental samples and which typically limit qPCR application.

Overall, our study reveals the inherent power of this approach for many other applications, including biomonitoring of key microbial functional groups for many natural and technological bioprocesses and microbiological surveillance.

## Figures and Tables

**Table 1:** Primers and probes utilized in this study for AOB and total bacteria quantification.

Primer/Probe	Sequence	Reference
CTO189fAB	5′-GGAGRAAAGCAGGGGATCG-3′	[16]
CTO189fC	5′-GGAGGAAAGTAGGGGATCG-3′	[16]
Rt1r	5′-CGTCCTCTCAGACCARCTACTG-3′	[16]
TMP1	5′-FAMCAACTAGCTAATCAGRCATCRGCCGCTC-3′-TAMRA	[16]
Bac1055F	5′-ATGGCTGTCGTCAGCT-3′	[17]
Bac1392R	5′-ACGGGCGGTGTGTAC-3′	[17]

**Table 2:** Summary of the samples utilized in this study. The total microbial biomass concentration is expressed as g of Volatile Suspended Solids (VSS) per liter of sludge for mixed microbial communities from engineered systems (AS; R1–R4) and as 16S rRNA gene copies L^−1^ of total bacteria from filtered water of environmental samples (SW and FW). AOB/total bacteria ratio (%) was calculated from qPCR data.

Sample	Source	Total Biomass	AOB/Total Bacteria
AS	Activated sludge from a municipal WWPT	2.0 g VSS/L	2.2%
R1	Laboratory-scale reactor for nitrogen removal	4.6 g VSS/L	6.3%
R2	Laboratory-scale reactor for nitrogen removal	4.2 g VSS/L	9.5%
R3	Laboratory-scale reactor for nitrogen removal	1.25 g VSS/L	8.6%
R4	Laboratory-scale reactor for nitrogen removal	1.2 VSS/L	12.6%
SW	Seawater	3.33 × 10^8^ gene copies/L	0.3%
FW	Freshwater	6.96 × 10^6^ gene copies/L	10.2%

**Table 3:** Mean, SD, CV, and linearity of the dilution series of the seven samples analyzed by qPCR with different chemistries, as reported in Figure 1. The mean value has been calculated by normalizing the values of each dilution point within the dilution series to the low-dilution one. Mean and SD are reported as log gene copies μL^−1^ reaction volume.

	SYBR Green				TaqMan			
Sample	Mean	SD	CV	R2	Mean	SD	CV	R2
AS	4.34	0.35	8.06	0.97	5.37	0.58	10.80	0.90
R1	4.53	0.05	1.10	1.00	4.63	0.13	2.80	0.99
R2	3.58	0.51	14.24	0.98	4.02	0.08	1.99	1.00
R3	4.80	0.14	2.91	1.00	4.57	0.06	1.31	1.00
R4	4.49	0.12	2.67	1.00	4.33	0.06	1.38	1.00
SW	2.76	0.10	3.62	0.99	n.d. ^1^	n.d. ^1^	n.d. ^1^	n.d. ^1^
FW	4.91	0.51	10.38	0.94	1.90	0.32	16.84	0.99

^1^ Data not reported. qPCR quantification was obtained only on two-fold diluted sample.

**Table 4:** Mean, SD, CV, and linearity of the dilution series of the seven samples analyzed by ddPCR with different chemistries, as reported in Figure 1. The mean value has been calculated by normalizing the values of each dilution point within the dilution series to the low-dilution one. Mean and SD are reported as log gene copies μL^−1^ reaction volume.

	EvaGreen				TaqMan			
Sample	Mean	SD	CV	R2	Mean	SD	CV	R2
AS	3.12	0.08	2.48	0.99	2.10	0.06	2.81	0.98
R1	2.34	0.02	0.65	0.99	2.08	0.30	14.47	0.98
R2	2.80	0.07	2.53	0.99	2.09	0.28	13.54	0.95
R3	2.30	0.06	2.54	0.98	2.02	0.10	4.72	0.99
R4	2.06	0.04	2.05	0.99	1.44	0.16	10.86	0.98
SW	2.23	0.02	0.75	1.00	1.55	0.09	5.69	0.98
FW	2.29	0.17	7.61	0.84	1.75	0.22	12.81	0.99

## Data Availability

The original contributions presented in this study are included in the article/Supplementary Material. Further inquiries can be directed to the corresponding author.

## Supplementary Materials

The following supporting information can be downloaded at https://www.mdpi.com/article/10.3390/microorganisms13112426/s1, Figure S1: Amplification bands at different annealing temperatures on 2% agarose electrophoresis gel. L1 and L7: DNA marker GeneRuler 100 bp DNA Ladder; L2: 60.4 °C; L3: 60 °C; L4: 59.4 °C; L5: 58.5 °C; L8: 57.4 °C; L9: 56.4 °C; L10: 55.8 °C; L11: 55.5 °C; L6 and L12: no template control; Table S1: Quantification range for each sample analyzed by ddPCR and qPCR; Table S2: Quantification of AOB by ddPCR as gene copies per volume of DNA extract. n = biological replicates performed in duplicate for ddPCR; Table S3: Quantification of AOB by qPCR as gene copies per volume of DNA extract. n = biological replicates performed in triplicate for qPCR.

## Abbreviations

The following abbreviations are used in this manuscript:

qPCR	Real-Time PCR
ddPCR	Droplet Digital PCR
AOB	Ammonia-Oxidizing bacteria
WWPTs	Wastewater Treatment Plants
