# Supplementary material for: Quantitative Estimation of Low-Abundance Targets in Engineered Systems and Environmental Samples: Comparative Study Between Droplet Digital PCR and Real-Time PCR
Source: Microorganisms. 2025 Oct 23;13(11):2426. doi: 10.3390/microorganisms13112426 (PMC12654130; doi:10.3390/microorganisms13112426)
Supplement: Supplementary file 1 [file microorganisms-13-02426-s001.zip › microorganisms-3930933-supplementary.pdf]

Supplementary Materials

Quantitative estimation of low abundance target in engineered systems and environmental samples: comparative study between droplet digital PCR and Real Time PCR

Alessia Ayala Alban[1, §], Barbara Tonanzi[1,2,§], Simona Crognale[1,2], Francesca Di Pippo[1], Simona Rossetti[1,*]

[1] Water Research Institute, National Research Council of Italy, CNR-IRSA, Via Salaria Km 29.300, Montero-tondo, 00015 Rome, Italy.
2 National Biodiversity Future Center (NBFC), Piazza Marina 61, 90133 Palermo, Italy.
* Correspondence: simona.rossetti@cnr.it
§ These Authors contributed equally to this work

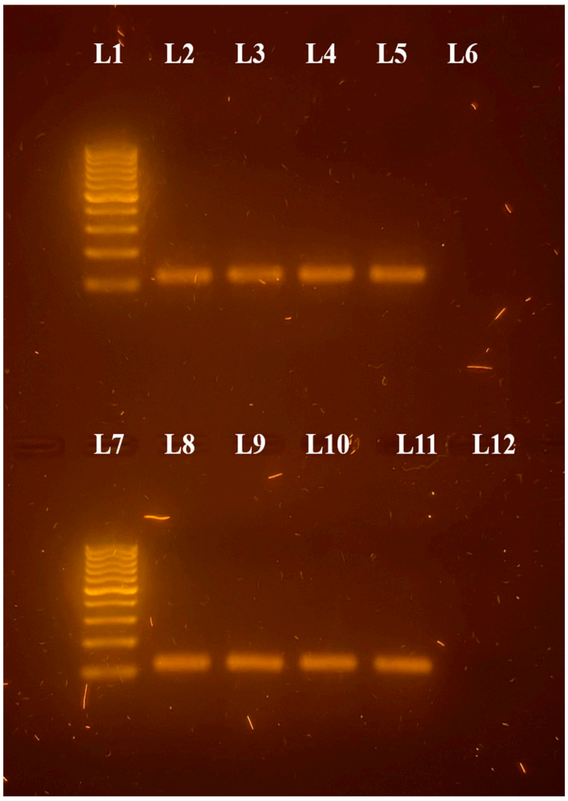

Figure S1. Amplification bands at different annealing temperatures on 2% agarose electrophoresis gel.
-L1 and L7: DNA marker GeneRuler 100 bp DNA Ladder;
-L2: 60.4°C; L3: 60°C; L4: 59.4°C; L5: 58.5°C; L8: 57.4°C; L9: 56.4°C; L10: 55.8°C; L11: 55.5°C;
-L6 and L12: no template control.

Table S1 Quantification range for each sample analyzed by ddPCR and qPCR.

Sample	qPCR SYBRGreen		qPCR TaqMan		ddPCR EvaGreen		ddPCR TaqMan	
	ng DNA	GC	ng DNA	GC	ng DNA	GC	ng DNA	GC
**AS**	1.3	4.65E+03	0.13	7.64E+02	0.03	15	0.16	26.3
	13	4.11E+04	130	8.85E+05	1.3	858	0.65	141.2
**R1**	0.4	3.34E+02	0.4	3.93E+02	0.04	21.9	0.05	3.29
	40	2.87E+04	40	3.10E+04	0.4	257	0.8	264.3
**R2**	0.24	1.66E+02	0.25	2.46E+02	0.04	16.6	0.026	1.0
	12	3.10E+03	12.7	8.15E+03	1.3	674.7	1.3	245.3
**R3**	0.41	7.80E+02	0.41	3.22E+02	0.04	20.39	0.05	4.87
	41	3.84E+04	41	4.14E+04	0.4	187.2	0.8	125.3
**R4**	0.88	9.91E+02	1.11	9.56E+02	0.025	16.14	0.025	2
	22	2.82E+04	22.3	2.51E+04	0.2	114.9	0.2	35.3
**SW**	0.6	7.18E+01	6	6.86E+02	0.6	17.4	0.6	4.69
	6	4.24E+02	-	-	6	162.9	6	29.3
**FW**	0.4	6.50E+03	0.92	7.52	0.46	30.4	0.46	18.36
	4.6	1.44E+04	4.6	1.66E+02	4.6	219.5	2.3	32.8

GC: gene copies μL^{-1} reaction volume.

Table S2 Quantification of AOB by ddPCR as gene copies per volume of DNA extract.
n= biological replicates performed in duplicate for ddPCR

Sample	Sample size (n)	ddPCR EvaGreen	Sample size (n)	ddPCR TaqMan
**AS**	5	2.55E+09 $\pm$ 4.62E+08	7	1.23E+09 $\pm$ 8.34E+08
**R1**	5	8.19E+08 $\pm$ 6.42E+07	3	1.69E+08 $\pm$ 2.0E+07
**R2**	5	2.34E+08 $\pm$ 4.99E+07	3	0.98E+08 $\pm$ 1.70E+07
**R3**	5	7.11E+08 $\pm$ 8.70E+07	6	3.10E+08 $\pm$ 8.90E+07
**R4**	5	4.07E+08 $\pm$ 4.88E+07	4	2.0E+08 $\pm$ 8.40E+07
**SW**	4	1.22E+06 $\pm$ 5.68E+04	4	0.80E+05 $\pm$ 8.06E+04
**FW**	4	7.47E+05 $\pm$ 2.84E+05	3	4.38E+05 $\pm$2.13E+05

Table S3 Quantification of AOB by qPCR as gene copies per volume of DNA extract.
n= biological replicates performed in triplicate for qPCR.

Sample	Sample size (n)	qPCR SYBRGreen	Sample size (n)	qPCR TaqMan
**AS**	3	0.54E+09 + 0.19E+08	2	3.7E+08 $\pm$ 2.0E+07
**R1**	4	1.70E+08 + 2.01E+07	4	2.02E+08 $\pm$ 3.69E+07
**R2**	3	3.6E+07 $\pm$ 1.8E+07	4	5.3E+07 $\pm$ 9.3E+06
**R3**	4	3.24E+08 $\pm$ 9.01E+07	4	1.72E+08 $\pm$ 3.68E+07
**R4**	3	1.59E+08 $\pm$ 4.76E+07	4	1.05E+08 $\pm$ 2.05E+07
**SW**	4	5.79E+05 $\pm$ 1.21E+05	2	1.25E+04 $\pm$ 1.5E+04
**FW**	3	1.77E+05 $\pm$ 9.57E+04	3	2.56E+04 $\pm$ 1.36E+04
